# Effect of Acute and Fractionated Irradiation on Hippocampal Neurogenesis

**DOI:** 10.3390/molecules17089462

**Published:** 2012-08-08

**Authors:** Min-Kyoung Park, Seolhwa Kim, Uhee Jung, Insub Kim, Jin Kyu Kim, Changhyun Roh

**Affiliations:** 1 Division of Biotechnology, Advanced Radiation Technology Institute (ARTI), Korea Atomic Energy Research Institute (KAERI), 1266, Sinjeong-dong, Jeongeup, Jeonbuk 580-185, Korea; Email: mkpark@kaeri.re.kr (M.-K.P.); shkim@kaeri.re.kr (S.K.); uhjung@kaeri.re.kr (U.J.); Insub@kaeri.re.kr (I.K.); jkkim@kaeri.re.kr (J.K.K.); 2 Department of Chemistry, Seoul National University, Gwanak-ro, Gwanak-gu, Seoul 151-747, Korea

**Keywords:** neurogenesis, ionizing radiation, immunohistochemistry, doublecortin, long-term effects

## Abstract

Ionizing radiation has become an inevitable health concern emanating from natural sources like space travel and from artificial sources like medical therapies. In general, exposure to ionizing radiation such as γ-rays is one of the methods currently used to stress specific model systems. In this study, we elucidated the long-term effect of acute and fractionated irradiation on DCX-positive cells in hippocampal neurogenesis. Groups of two-month-old C57BL/6 female mice were exposed to whole-body irradiation at acute dose (5 Gy) or fractional doses (1 Gy × 5 times and 0.5 Gy × 10 times). Six months after exposure to γ-irradiation, the hippocampus was analyzed. Doublecortin (DCX) immunohistochemistry was used to measure changes of neurogenesis in the subgranular zone (SGZ) of the hippocampal dentate gyrus (DG). The number of DCX-positive cells was significantly decreased in all acute and fractionally irradiation groups. The long-term changes in DCX-positive cells triggered by radiation exposure showed a very different pattern to the short-term changes which tended to return to the control level in previous studies. Furthermore, the number of DCX-positive cells was relatively lower in the acute irradiation group than the fractional irradiation groups (approximately 3.6-fold), suggesting the biological change on hippocampal neurogenesis was more susceptible to being damaged by acute than fractional irradiation. These results suggest that the exposure to γ-irradiation as a long-term effect can trigger biological responses resulting in the inhibition of hippocampal neurogenesis.

## 1. Introduction

Neurodegeneration, the progressive loss of structure or function of neurons, results in many neurodegenerative diseases such as Parkinson’s, Alzheimer’s, and Huntington’s [1,2,3,4]. The hippocampus, where neuronal circuitry ranging from molecular to systemic exists, has been recognized as a key region related to many neurological diseases [5,6,7]. Doublecortin (DCX) as a progenitor neural cell marker, a microtubule-associated phospho-protein, has been utilized for analyzing alterations in neurogenesis in the adult dentate gyrus (DG) because it is believed to be specific to neuronal antigens expressed by newly born neurons [8]. The rate of hippocampal neurodegeneration is known to be affected by age, hormonal status, growth factor, chemicals, physiologic stimuli and environmental effects. For many reasons, there is an increasing interest in models used to understand the mechanism of neurodegeneration [9,10,11,12].

Ionizing radiation has been hazardous to human health due to overexposure from natural and artificial sources, such as diagnostic and therapeutic medical usage [13,14]. Overexposure to radiation causes damage to living tissue, and can result in mutations, radiation sickness, cancer, and death [15,16]. Even though the risk to radiation exposure in the brain is a big issue, the changes in the hippocampal neurodegeneration are poorly understood. However, recently, the risk of short-term neurodegeneration caused by radiation exposure has been reported [17,18,19,20,21,22]. Kim’s group suggested that transient impairment of the functioning of the hippocampus is linked to inhibition of hippocampal neurogenesis after acute γ-irradiation [9]. The Abdallah group reported that irradiation can reversibly alter proliferation, neurogenesis, and cell death in the dentate gyrus of adult mice. The early cognitive deficits may be related to the effects of a short-term reduction of neurogenesis [17]. The Acevedo group reported that older female mice deficient in apoE (Apoe^−/−^) are more susceptible to effects of gamma irradiation on novel location recognition [23]. 

In this study, we addressed the long-term effect of γ-irradiation exposure on neurogenesis in a C57BL/6 female model. We were interested in determining if there is a dose response relationship in terms of radiation-induced long-term impairments in neurogenesis. We elucidated the number of DCX-positive cells on the hippocampus with altered radiation dose in immunohistochemistry. We hypothesized that the long-term changes in DCX-positive cells caused by γ-irradiation would continuously increase neurodegeneration resulting in damage to the hippocampal function as opposed to the recovering tendency of neurogenesis impairment in the short term. Understanding how irradiation affects neurogenesis may provide useful insight into potential approaches to reduce brain-related diseases.

## 2. Results and Discussions

**Figure 1:**
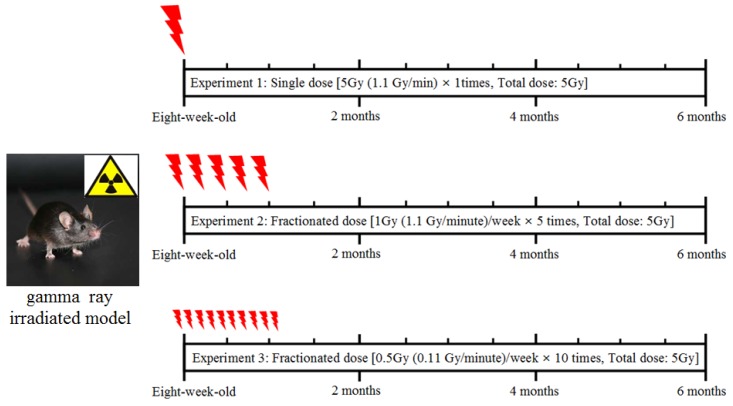
Gamma-ray irradiated models by acute or fractionated whole-body irradiation.

Figure 1 shows the scheme for the procedure of radiation exposure in an animal model. The mice were exposed to whole-body radiation at acute dose (5 Gy) or fractional doses (1 Gy × 5 times or 0.5 Gy × 10 times), and further sustained for 6 months.

**Figure 2:**
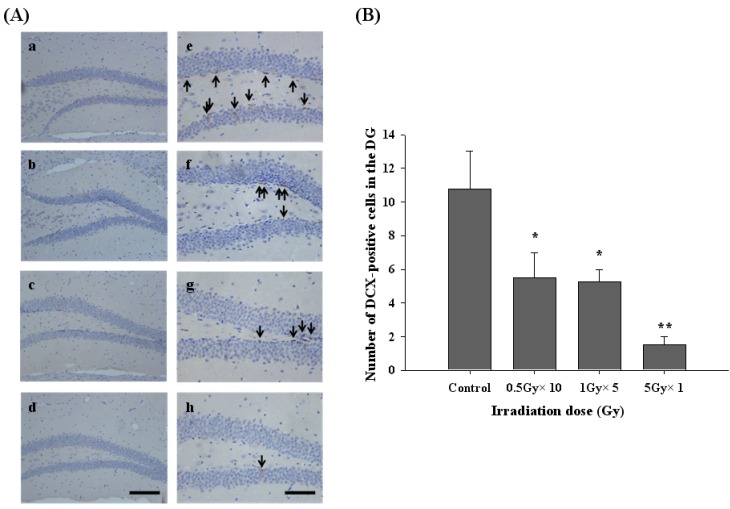
Long-term effect of acute and fractionated irradiation in subgranular zone (SGZ) of the hippocampal dentate gyrus (DG). (**A**) Histological results in images (**B**). The quantitative values of DCX-positive cells. Immunohistochemistry of DCX in the DG of the hippocampus in four groups of mice. Comparison profiles of DCX expression of the control (a and e), 0.5 Gy × 10 times (b and f), 1 Gy × 5 times (c and g), 5 Gy × 1 times (d and h) groups. The bar graphs indicate the number of DCX-positive cells/DG (mean ± SD).The arrows were marked. Scale bars = 100 μm (a–d), 50 μm (e–h). * *p* < 0.05, ** *p* < 0.005.

To investigate the long-term effect of radiation exposure on hippocampal neurogenesis in an adult mouse DG, the number of DCX-positive cells in the subgranular zone (SGZ) of the hippocampal DG was analyzed by using immunohistochemistry (Figure 2). Six months after radiation exposure, the number of DCX-positive cells in the hippocampal DG of the control group was 10.75 ± 2.25 cells/DG (Figure 2A, a and e). In the fractional (0.5 Gy × 10 times and 1 Gy × 5 times) and acute irradiation (5 Gy × 1 times) groups, the numbers of DCX-positive cells were 5.5 ± 1.5 cells/DG (Figure 2A, b and f), 5.25 ± 0.75 cells/DG (Figure 2A, c and g) and 1.5 ± 0.5 cells/DG (Figure 2A, d and h), respectively. The groups of fractionated irradiation (0.5 Gy × 10 times and 1 Gy × 5 times) showed approximately 49% and 51% reductions of DCX-positive cells in the hippocampal DG compared to the control group. Importantly, the group with acute irradiation (5 Gy × 1 times) resulted in an 86% reduction of DCX-positive cells. 

DCX is a reliable marker of newly generated neurons because neuronal precursor cells begin to express DCX while actively dividing [8,24]. DCX immunoreactivity, which quantifies both the absolute number and dendritic growth of new neurons, can be useful for analyzing the modulation of hippocampal neurogenesis as a function of neurodegenerative diseases. In this study, it was shown that the number of DCX-positive cells by acute or fractionated irradiation groups tended to be significantly reduced (Figure 2B). Thus, the results of DCX immunoreactivity suggests that the long-term effect of γ-irradiation exposure can suppress hippocampal neurogenesis resulting in damage to the hippocampal function unlike the tendency to fully restore transient impairments through the short-term effect of irradiation in previous studies [17,18].

Radiation therapy plays a major role in the treatment of primary brain tumors and cancers. Unfortunately, however, radiation exposure can cause severe side effects including cognitive deficits. According to recent studies, irradiation effects in normal brain tissue have been principally limited to studies of acute dose irradiation [25]. Our goal in this study was to characterize the long-term effects by acute and fractionated irradiation on hippocampal neurogenesis. DCX immunoreactivity was relatively lower in the acute irradiation group than in the fractionated irradiation groups, but there was no significant difference in the fractionated irradiation (Figure 2B). These results showed that hippocampal neurogenesis was more vulnerable to acute than fractionated irradiation.

## 3. Experimental

### 3.1. Chemicals and Mice

Six-week-old female C57BL/6 mice were obtained from Orient Bio Inc. (Seongnam, Gyeonggi-Do, Korea), and housed in standard conditions under a temperature of 23 ± 1 °C and humidity of 55 ± 5% with a 12-hour light-dark cycle. After two weeks of adaptation, the mice were divided into four groups with three mice in each group. All mice had free access to standard food and water. All procedures were conducted under guidelines for the use and care of laboratory animals at Korea Atomic Energy Research Institute (KAREI). All reagents were of the highest grade available.

### 3.2. Ionizing Radiation in Mice

Eight-week-old mice were exposed to whole-body γ-irradiation with γ-rays from a 137Cs source (Gammacell 40, Nordion International Inc., Ottawa, ON, Canada) without anesthesia [26,27]. The long term effect of γ-irradiation on neurogenesis was observed after whole-body irradiation in the mice with acute 5 Gy (1.1 Gy/min) dose or fractionated doses (1 Gy [1.1 Gy/min]/week, 5 times and 0.5 Gy [0.11 Gy/min]/week, 10 times). Fractionated irradiation was delivered to the whole-body of the mice at a 5 Gy cumulative dose for five weeks (Figure 1).

### 3.3. Immunohistochemistry

The mice were sacrificed and their brains were dissected (n = 3 mice/group). The brains were processed for embedding in paraffin wax after fixation in 10% neutral buffered formalin using routine protocols for immunohistologic evaluation. For the DG area in the hippocampus, the brain of each mouse was sampled approximately 2.22 mm behind the bregma. In addition, 5 μm-thick coronal sections were cut by microtome (RM2145; Leica, Nussloch, Germany). A standardized counting area, five sections from a total of one-hundred sections, or one out of twenty, serial sections, were analyzed for immunohistochemistry. As the next step, the samples were deparaffinized by routine protocols, exposed to a citrate buffer (0.01 M, pH 6.0; S2031, Dako, Hampshire, UK) and heated in an autoclave for 10 min. After heating, the slides were allowed to cool for 20 min. All subsequent steps were performed at room temperature. The sections were treated with a peroxidase block (K4003 Kit, Dako) for 30 min to block endogenous peroxidase activity. The sections were treated with a protein block (X0909, Dako) for 20 min and then incubated for 1 h with a polyclonal rabbit anti-DCX antibody (diluted 1:500; ab18723, Abcam Antibodies, Cambridge, UK). They were then reacted with goat anti-rabbit immunoglobulin conjugated with peroxidase labeled polymer (K4003 Kit, Dako) for 30 min. The peroxidase reaction was developed using a diaminobenzidine substrate buffer (K4003 Kit, Dako) according to the manufacturer’s instructions for 30 sec. After the completion of color development, the sections were counterstained with hematoxylin (S3309, Dako) for 5 min, washed in running tap water for 20 min, dehydrated through a graded ethanol series, cleared with xylene, and mounted with permount (SP15-500, Fisher Scientific, Pittsburgh, PA, USA).

### 3.4. Statistics

The number of DCX-positive cells, which are immature progenitor cell markers in the hippocampus, was scored. A statistical analysis was performed using a one-way analysis of variance (ANOVA). The values are expressed as average ± standard deviation. The *p* values were considered as significant. 

## 4. Conclusions

In conclusion, this is the first report to elucidate the long-term effects of DCX-positive cells as an immature progenitor cell marker by acute and fractionated irradiation on hippocampal neurogenesis *in vivo*. To the best of our knowledge, no data exist with respect to the radiation response of acute and fractionated irradiation on hippocampal neurogenesis in animal model. This is important because the effect of brain treatment in acute irradiation is higher than that seen in fractionated irradiation. The long-term effects of irradiation are important in clinic because of chronic progressive nature and significant long-term morbidity.
